# Massive anorectal abscess caused by rectal cancer

**DOI:** 10.1002/ccr3.6388

**Published:** 2022-10-11

**Authors:** Risa Hirata, Masaki Tago, Hidetoshi Aihara

**Affiliations:** ^1^ Department of General Medicine Saga University Hospital Saga Japan

**Keywords:** anorectal abscess, computed tomography, pelvic abscess, rectal cancer

## Abstract

A 66‐year‐old man with perianal pain was found to have a tender erythematous mass on the left side of the anus. Thoracoabdominal computed tomography with contrast enhancement showed a massive anorectal abscess extending from the rectum to the perianal area. The final diagnosis was anorectal abscess caused by rectal cancer.

## Abstract 2

Rectal cancer, the incidence of which has been increasing recently, can cause anorectal abscesses. Physical examination, endoscopy, and computed tomography are required to identify the cause of an anorectal abscess.

A previously healthy 66‐year‐old man who had lost 7 kg in the previous 6 months presented with diarrhea and perianal pain for 3 weeks. His body temperature was 38.4°C, blood pressure 112/75 mmHg, and pulse rate 120 beats/min. No abnormalities were found on abdominal examination; however, a tender erythematous mass was found on the left side of his anus. Blood examination showed a white blood count of 15.9 × 10^9^/L and C‐reactive protein of 1866 nmol/L. Thoracoabdominal computed tomography (CT) with contrast enhancement revealed fistulas, soft tissue swelling, and a massive, encapsulated, gas‐forming, anorectal abscess from the rectum to the perianal area (Figure 1). Subsequent colonoscopy and histopathological examination resulted in a diagnosis of well‐differentiated tubular adenocarcinoma extending from the rectum to the anal canal. Antimicrobial therapy and surgery improved his condition.

**FIGURE 1:**
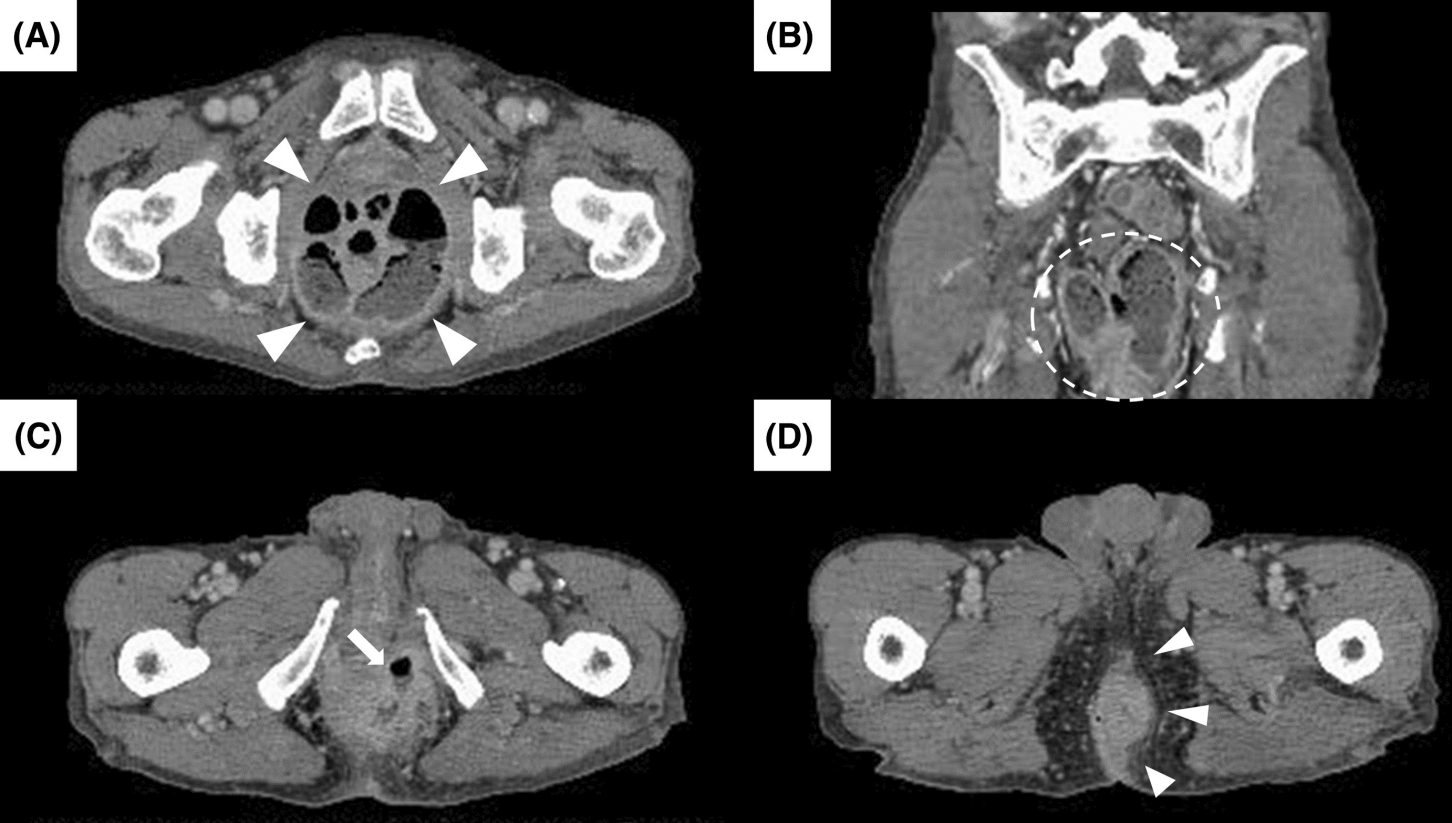
Thoracoabdominal contrast‐enhanced computed tomography (CT) images (A, C, D): horizontal sections; (B): coronal section. CT revealed a massive, encapsulated abscess with soft tissue shadows and air from the rectum to the perianal area ((A), arrowheads; (B), dotted circle, anal fistula at 2 o'clock (C), arrow), and swollen soft tissue on the left side of the anus ((D), arrowheads).

Anorectal abscesses are caused by non‐specific obstruction and infection of the glandular crypts, hemorrhoids, Crohn disease, trauma, and malignancy.^1^ Although CT is useful for diagnosing anorectal abscesses, it can be difficult to detect their cause. A comprehensive diagnosis therefore requires physical examination, including examination of the perianal and rectal regions, and colonoscopy, especially for patients with chronic weight loss.^1^ Given that the prevalence of colorectal malignancies has been increasing in Japan,^2^ physicians should check carefully for rectal malignancies in patients with anorectal abscesses.

## AUTHOR CONTRIBUTIONS

RH was involved in the literature search, study conception, and drafting of the manuscript. MT was involved in the literature search, study conception, and drafting and revision of the manuscript. HA was involved in the literature search, study conception, and clinical care of the patient.

## FUNDING INFORMATION

There is no funding for this article.

## CONFLICT OF INTEREST

The authors state that they have no conflict of interest.

## ETHICAL APPROVAL

This manuscript conforms to the provisions of the Declaration of Helsinki in 1995 (as revised in Brazil 2013).

## CONSENT

Written informed consent was obtained from the patient to publish this report in accordance with the journal's patient consent policy.

## Data Availability

The data that support the findings of this study are available from the corresponding author upon reasonable request.
